# Latino Adults’ Perspectives on Treating Tobacco Use Via Social Media

**DOI:** 10.2196/mhealth.6684

**Published:** 2017-02-08

**Authors:** Beatriz Anguiano, Cati Brown-Johnson, Lisa G. Rosas, Cornelia Pechmann, Judith J. Prochaska

**Affiliations:** ^1^ Stanford Prevention Research Center Department of Medicine Stanford University Stanford, CA United States; ^2^ Palo Alto Medical Foundation Research Institute Palo Alto, CA United States; ^3^ The Paul Merage School of Business University of California, Irvine Irvine, CA United States

**Keywords:** smoking cessation, tobacco, Latino, Hispanic, social media, focus group

## Abstract

**Background:**

Latinos are the largest minority group in the United States, and in California they outnumber non-Hispanic whites. Smoking cessation programs tailored for Latino culture, and this population’s specific smoking patterns, are needed. Online social networks for smoking cessation have high potential for Latinos, but have not been tested to date.

**Objective:**

Building a research program on social media apps for cancer prevention in diverse populations, this qualitative study assessed acceptability of tobacco treatment that was distributed via social media for Latino smokers.

**Methods:**

We conducted three focus groups with Latino adults who were former and current smokers recruited from Santa Clara County, California in 2015 (N=32). We assessed participants’ smoking histories, attempts to quit, social media exposure, and receptivity to a social media-based smoking cessation intervention. Audio transcripts were translated and coded for themes.

**Results:**

Participants reported factors driving their tobacco use and motivations to quit, and emphasized the importance of community and family in influencing their smoking initiation, cravings and triggers, attempts to quit, and abstinence. Participants valued the communal aspect of social media and suggested strategically tailoring groups based on key features (eg, age, gender, language preference). Participants reported preferring visual, educational, and motivational messages that were connected with existing services.

**Conclusions:**

Participants generally voiced acceptability of a social media-delivered intervention to help them quit smoking, viewed the intervention as well-equipped for catering to the strong community orientation of Latinos, and suggested that the platform was able to address variation within the population through strategic group creation. As a group member reflected, “Podemos hacerlo juntos” (We can do it together).

## Introduction

Tobacco is the leading preventable cause of death, and is linked to a dozen types of cancer, along with heart and lung disease [1]. Traditional smoking cessation programs remain underutilized, particularly among racial and ethnic minorities such as Latinos [2]. Latinos are the largest racial/ethnic minority in the United States and in some areas, such as California, Latinos outnumber non-Hispanic whites (15.19 million vs 14.88 million) [3]. Latino smokers tend to smoke fewer cigarettes than non-Hispanic white smokers and are less likely to be daily smokers than smokers of all other racial/ethnic groups; hence, cessation pharmacotherapy may not be indicated [4]. Furthermore, research indicates that light and intermittent Latino smokers are infrequently advised to quit by healthcare professionals [5]. Among Latino smokers surveyed at a community health fair, only 5% reported ever using cessation medications, and less than 6% were aware of smoking quit-lines [6]. However, surveys of light and intermittent Latino smokers have indicated high readiness to quit and interest in smoking cessation programs [7]. Given light and intermittent smoking patterns and low penetrance of existing smoking cessation programs, innovative behavioral or psychological approaches are needed for the large population of Latinos in the United States.

Tailoring of cessation treatment strategies to target audiences has been a strategy for increasing reach and engagement. In the early 1990s, a printed Spanish-language smoking cessation guide was found to support quitting and was distributed as a best practice [8]. A 2003 review of the research literature identified 10 published tobacco treatment studies that were targeted to Latino smokers, some of which included video and audio enhancements [9]. The authors concluded that greater innovations were needed to leverage state-of-the-art practices for treating tobacco addiction in ethnic minorities, with a focus on Latino smokers [9]. A 2011 review identified an additional 5 tobacco treatment studies focused on Latino smokers; added to Spanish language print materials were home visits with lay health advisors, telephone counseling, and group sessions [10]. Findings generally indicated that treatments increased abstinence, at least in the short-term [10]. The need for more research, with a particular focus on Latino smokers, was underscored. Herein, we sought to explore whether cessation treatments could be acceptably delivered via virtual support groups on mobile devices.

Online social media sites allow real-time interactivity and peer-to-peer support, which may build upon cultural norms and values, with potentially low-cost application for disseminating health interventions to diverse groups. Furthermore, communications generated and catalogued on social media sites provide novel information for better understanding transitions in smoking and emerging product use (eg, electronic cigarettes; e-cigarettes). Twitter is the dominant open social media site, with a reported 320 million active monthly users, representing growth of 9.6% over the same period a year prior [11]. As a platform, Twitter has been utilized in over 140 medical and health care applications [12]. When studying the treatment of tobacco use in a randomized controlled trial (N=160), we found self-reported sustained abstinence for 60-days was 40% for a Twitter smoking cessation support group versus 20% for the comparison group (*P*=.012); 81% of the sample was non-Hispanic white [13]. To support broader reach and engagement, evidence of acceptability among Latino smokers is needed.

Social media is likely to be a viable platform for Latino adult smokers, given the widespread use of the Internet, particularly for the dissemination of health information. A 2010 Pew Hispanic Center study reported that 83% of Latinos received health information from media sources, including 35% online [14]. Furthermore, 64% reported having changed their behavior based on information from online health sources [14]. From 2009 to 2013, Pew data indicated that Latinos in the United States crossed the *digital divide*, exceeding non-Hispanic whites in cellphone ownership (86%), going online from a mobile device (75%), and social networking (68%) [15]. Furthermore, Latino consumers share information via social media fivefold more often than non-Latino users [15]. Among Latinos in the United States who access social media, 60% do so in English, 29% in Spanish, and 11% equally using English and Spanish; by nativity, 86% of Latinos born in the United States use English, while 55% of foreign-born Latinos prefer Spanish [15]. Within a context of low access to health care, high social media use, and differing language preferences, electronic health approaches have been suggested as ideal methods for reaching Latinos [16]. However, the use of online social networks to aid Latino adults with smoking cessation has not been tested.

To inform a social network-based smoking cessation program, we conducted focus groups with Latinos who were current and former smokers to determine if a tobacco cessation treatment distributed via social media would be acceptable. Secondary research aims were: gathering information and feedback about local Latino smoking profiles, attempts to quit, and social media exposure to inform the intervention strategy and community outreach efforts.

## Methods

### Sample

Participants were recruited via online classified advertisements (Craigslist), in person by community health workers, and through word-of-mouth in Santa Clara County. Inclusion criteria were: age 18 years or older; identifying as Latino/Latina; residing in the Santa Clara County area; and status as a current daily, social, or former smoker.

### Procedures

The focus group moderator was fluent in Spanish and English. The groups were semistructured. The moderator guide prompted questions about mobile phone and social media use, smoking, quitting smoking, and treatment preferences. An initial survey assessed participants’ demographic and smoking history information. Study procedures were approved by the Stanford Institutional Review Board; all participants provided signed informed consent in Spanish or English, were compensated US $50 for their time, and received a meal during the focus group session.

### Data Reduction and Analysis

Each focus group was audio recorded. A Spanish/English bilingual coder listened to the focus group audio recordings and outlined initial coding themes, which were discussed and refined by the research team. The audio recordings were simultaneously translated and transcribed to a final written transcription in English. Using a detailed codebook, the same bilingual team member then coded the written transcripts for emergent and preidentified themes of interest using Dedoose [17]. A second coder utilized the codebook to review the coding of the written transcripts. Discrepancies were discussed with the senior author to come to consensus.

## Results

### Sample Description

A total of 32 individuals (15 men, 17 women) from Santa Clara County, California participated. Participants included 19 current daily smokers, 4 intermittent or nondaily smokers, and 9 former smokers. Daily smokers averaged 8.4 cigarettes per day (standard deviation [SD] 10.4, range 1-40) and nondaily smokers averaged 4.3 cigarettes per week (SD 3.8, range 1-8). Current smokers reported time to first cigarette upon waking within 5 minutes (3/23, 13%), between 6-30 minutes (3/23, 13%), between 31-60 minutes (6/23, 26%), and greater than 60 minutes (11/23, 48%). Factors that kept participants from smoking sooner included children, having to go outside to smoke, TV, and checking Facebook. Participants reported getting their cigarettes from friends (n=15), gas stations (n=13), liquor stores (n=12), and corner stores (n=12).

All participants had made at least one 24-hour attempt to quit smoking (range 1-7). Identified reasons for quitting related to money, work, a home smoking ban, family and friends, cancer fears, sports, and not feeling the urge to smoke. Among the 23 current smokers, 6 (26%) were not intending to quit in the near future (precontemplation), 7 (30%) intended to quit in the next six months (contemplation), and 10 (43%) were planning to quit in the next month (preparation). Three individuals reported assistance for quitting smoking from a medical provider. Only one respondent reported using nicotine replacement. No participants reported using other cessation medications or formal psychosocial supports to quit (eg, group or individual counseling, quit-line).

Most participants owned a smartphone (27/32, 84%), and all but one kept their phone with them every day. The majority of respondents reported having their phone turned on all the time (20/32, 63%), texting on their phone more than once daily (26/32, 81%), and checking their Facebook page at least once daily (22/32, 69%).

### Tobacco Use Association and Triggers

At the start of the focus groups, in a word association task (ie, “What word comes to mind when you think of smoking?”), participants connected smoking to negative health and social effects in the following order of frequency: cancer, money, aging skin, and guilt. Participants also identified positive aspects of smoking, including social activity, calming, weight loss, and hobby.

Triggers for smoking were mentioned throughout the focus group conversations. In order of frequency (with counts indicated) participants identified: stress from school, work, family, and traffic (11); negative emotions such as anger and anxiety (6); alcohol use (4); other habitual triggers (4); others smoking (3); work breaks (2); boredom (2); seeking relaxation (2); and smoking for gastrointestinal regularity (1). Notably, social media was not identified as a trigger to smoke.

### Motivations for Quitting Smoking

While a minority of participants were former smokers, all had experience with quitting for at least 24-hours. Motivations for quitting centered around family, including children, siblings, partners, and parents:

I would hide my cigarettes, I used perfume so that my son couldn’t smell the cigarette, I would wash my hands, but on one occasion he looked at me and he said, “Oh, you’re smoking!” I felt like a bucket full of water fell over me, he said, “Do you want to die? If you don’t care about me, continue smoking.” His words hurt me so much that in 15 days I quit because I thought that a cigarette was not more important than my son. It was very hard, I had terrible headaches, shaking… but the love for my son is what helped me quit smoking.”

Life transitions were a common theme, overlapping with family concerns, as pregnancies and new babies were prominent transitions. Two women and a man successfully quit smoking during a family pregnancy. As one woman shared:

*I started smoking when I was 13, and I quit smoking when I was 41, because I got pregnant. After 28 years, it was very difficult for me to quit smoking, but it was the promise I made because I got pregnant, and I haven’t smoked for 15 years.*

Another woman described her shame and concerns around not being able to quit during pregnancy:

My last pregnancy - I did smoke. It caused me a lot of pain, and I have four children with asthma because I smoked when I would breastfeed. My youngest girl also has asthma. I’ve always had bronchial disease, and my kids would tell me, “I don’t want you to die.” I knew it was wrong, but I would get mad, or I’d get sad, and I would get out to smoke. Sometimes I get an urge to smoke, but I love my children a lot, and I want to live for them.

Additional influences identified as motivating cessation were religious faith, medical advice, and financial and health concerns. One participant said her sister became a Christian and stopped smoking, while another shared her promise to God to quit smoking. A third participant shared, “I always would ask my God, ‘You know what? I can’t do this alone, help me to give up this obsession.’”

### Social Support and Community

The importance of social support and community were identified as themes. Participants noted that *two heads think better than one* and emphasized a shared belief that humans are social beings. Another explained, “Sometimes we only need support... you can succeed because there’s somebody who wants the same for you.” The salience of broader community support was particularly relevant in the context of the isolation of immigration. One participant talked about a friend who confided that she only smoked because she was lonely and sad as an immigrant. She shared:

Sometimes people need to be in a group to be able to see how other people are trying hard to quit in order to encourage themselves to quit too. Some people are in this country and they are alone, so I think [support] would help them.

### Social Media for Quitting Smoking

#### Platforms

Participants reported using various social media platforms, including Facebook, Twitter, Instagram, Skype, Yahoo, and Snapchat. The groups stated a strong preference for Facebook and visual messages. The overall sentiment across groups was acceptability of social media as a vehicle for smoking cessation programs. One participant stated, “I think this idea is very good because… we’re 100% cybernetic… and [social media] is the right weapon to use.” Another asserted, “I think it would be a magnificent idea because… I have people on there [Facebook] that put they feel bad… and we send them a message, and it helps.” Furthermore, social media smoking cessation groups were imagined as supportive of quitting, in contrast to existing social networks of smokers who may encourage continued smoking. Participants also liked the idea of knowing that strangers in a group would withhold judgment toward any failed quit attempts.

Not all participants agreed, however, with one participant stating that his family and friends would be better able to support his quitting compared to, “a group of strangers.” One participant resisted the idea of spending more time on her phone, noting that as a parent her time at home is already too hectic. Another respondent voiced concern, explaining:

*I don’t know how comfortable people would be about going into a group… share with someone that they don’t know. Being anonymous… especially since Latinos… we tend to be more like, who do I know versus I don’t know you. I think that might become an issue*.

#### Group Formation

Participants discussed whether the groups should be matched on salient characteristics. One social smoker wanted to be in a group of nondaily social smokers. Another participant suggested creating groups based on common interests, similar to what is done on “dating sites.” A discussion centered on matching participants by age. One young adult participant stated, “If I see a young person trying to stop… we can do it together.” Another participant voiced potential benefits of mixed-age groups, stating:

different ages could help. I know that for the young ones, the pressure they have is very difficult, even more if they’re in school, they get stressed out, and they want to relax. So having an adult in that group who has more knowledge could be beneficial to them.

Other respondents encouraged the idea of keeping age unknown. One participant, with the perspective that age should not matter, asserted, “Cancer doesn’t look at ages, or race.” Another participant reflected:

*The most convenient thing is to have the ages unknown because maybe the one who is smoking really needs help. If they say, ‘Oh, it’s a person who is 60,’ and they’ll say, ‘What’s this old man or woman going to know?’*

#### Language Preference

Regarding language preference for a Latino-focused smoking cessation intervention on social media, 7 participants preferred a mixed English/Spanish platform, 4 preferred Spanish only, and 1 preferred English only. Three additional participants did not have a preference, and opted for group leaders to choose. The other 17 participants did not voice a preference. All participants, except the one who preferred English only, reported that Spanish was their dominant language.

#### Messaging

Participants shared advice on the types of smoking messages that would be most effective for individuals trying to quit smoking. There was a preference for nonforceful communication with no demanding messages, such as, “Don’t push; we’ll do it because we want to do it.” Preferred messages were educational and provided motivation and support. One participant emphasized, “It’s important that we know why we are making the decision to quit. It’s good to help us understand why we made decisions to quit.” Participants also recommended linking social media cessation interventions with existing support systems and services, such as the national smokers quit-line (1-800-QUIT-NOW).

The use of visual images was also encouraged, reflected by the quote, “A picture has a bigger impact than a word.” One participant shared, “I have a friend and he’s a doctor and he continuously post lungs… [and information] about cigarette filters.” Two participants stated that it was uncommon to see images or information about smoking on social media, while others noted postings of drinking and smoking at parties rather than encouragement to quit.

### Electronic Cigarettes

Despite not being part of the discussion guide, e-cigarettes represented an emergent topic with a variety of expressed opinions. Some participants were positive towards e-cigarettes with assertions that, “They’re not as bad as cigarettes”, “They’re cheaper than tobacco”, and not a “bother” to others with a bad smell. Participants reported seeing e-cigarette advertising cessation claims, although no respondent reported successfully quitting smoking using e-cigarettes. Participants reported a willingness to try e-cigarettes, largely out of curiosity instead of a desire to quit smoking.

## Discussion

Latino smokers and recent former smokers from the Bay Area of California largely found the concept of a social media tobacco cessation intervention acceptable. Social media was perceived to be well-equipped to meet the social- and community-oriented experiences of Latinos. Participants also noted that social media could allow for further tailoring of support groups based on homophilous characteristics related to age, smoking frequency, and language preference. A preference was stated for Facebook, due to participant familiarity with the platform and the ability to leverage visual as well as text-based content. Visual communication of health information improves comprehension, and enhances attention, memory, and recall [18]. Our team’s recent evaluation of *Tweet2Quit* in a largely non-Hispanic white sample found that participant engagement (ie, tweeting) predicted success in quitting smoking [13]. More visual messages, as attention-attractors, may encourage quitting success via increased engagement.

Focus groups have been used in research to explore the experience of smoking cessation among ethnic minorities and have highlighted the importance of considering levels of acculturation in program tailoring [19]. Although our groups did not expressly discuss acculturation, participants highlighted a desire for social media smoking cessation groups constructed around similar age and language preferences, which are two potential indicators of acculturation.

Family orientation, social support, and community were prevailing themes in the focus group discussions of smoking, attempts to quit, and social media use, providing a basis for why social media may be particularly well-suited for a Latino-focused smoking cessation intervention. Participants noted the opportunities for community-building in social media venues, which have not previously been available through traditional websites or quit-line interventions. The Latino experience of quitting smoking is also conceptualized as a family or group effort, and social media may address previous calls to tailor interventions for racial/ethnic-specific processes for quitting.

As a local qualitative study, the generalizability of our results is limited. The group moderator was fluent in Spanish, and coding was done via listening in Spanish; however, the final analysis of transcripts was conducted in English, which may have reduced or changed content in unpredictable ways. Despite these limitations, findings with respect to acceptability of social media, importance of family in health behavior change, and preference for visual material are likely broadly relevant.

In conclusion, a social media-delivered intervention to support smoking cessation appears to be acceptable for Latino smokers. Regarding immediate implications, the study findings support efforts to develop novel interventions for treating tobacco use via social media. These interventions may be tested as standalone cessation programs or as adjuncts to existing treatments. For cultural relevance, message themes within the program should attend to family and community ties and influences. For maximum engagement and inclusiveness, flexibility in language use (ie, English, Spanish, both) should be permitted and encouraged. The specific social media platform may be determined by usage rates and fit of the technology for the intervention’s approach and privacy concerns. Regardless of platform, community outreach and engagement is essential to treatment impact, and Latino smokers’ tobacco purchasing behaviors may inform channel selection. Based on the focus groups, places to promote a social media quit smoking program would include in gas stations, liquor stores, and corner stores, near where cigarettes are displayed, as well as via word-of-mouth referrals from friends.

The use of social media by Latinos is high; however, the use of these media for health behavior change appears to be underdeveloped. As such, our next steps will center on developing and testing a Latino-specific, bilingual, private, support group-based social media intervention for smoking cessation. The examination of homophily in group communications will be of particular interest, to determine whether directed and reciprocated communications align around shared member characteristics (eg, gender, age, daily/nondaily smoking status, language preference).

## Abbreviations

e-cigarette: electronic cigarette
SD: standard deviation
